# The impact of SARS‐CoV‐2 mRNA vaccine on intracytoplasmic sperm injection outcomes at a fertility center in Iraq: A prospective cohort study

**DOI:** 10.1002/hsr2.2142

**Published:** 2024-05-23

**Authors:** Hawraa Sahib Al‐Haddad, Hayder A. L. Mossa, Amal Abdulwahid Muhammed, Alaa Salah Jumaah, Katherine Ann McAllister, Akeel Abed Yasseen

**Affiliations:** ^1^ Al‐Nahrain University Baghdad Iraq; ^2^ Department of Pathology and Forensic Medicine, Faculty of Medicine University of Kufa Iraq; ^3^ School of Biomedical Sciences Ulster University Northern Ireland UK

**Keywords:** fertilization rate, ICSI, mRNA vaccination, oocyte quality, SARS‐CoV‐2

## Abstract

**Background and Aims:**

Coronavirus disease 2019 (COVID‐19) is a major public health problem that requires preventative vaccines. However, there is vaccine hesitancy among women of reproductive age in Iraq. This study aimed to investigate SARS‐CoV‐2 vaccination effects on intracytoplasmic sperm injection (ICSI) and related fertility parameters.

**Methods:**

The study population comprised 54 infertile patients undergoing the ICSI procedure at a fertility clinic: vaccinated (*n* = 17) and non‐vaccinated (*n* = 37). SARS‐CoV‐2‐IgG/mL was assayed in follicular fluid from patients. Fertility parameters were assessed using oocyte and embryo quality and pregnancy outcomes between study groups, with respect to the time interval from vaccination to ova pick up.

**Results:**

There were no significant differences between non‐vaccinated and vaccinated groups in respect of oocytes quality with regard to the mean number of picked up oocytes (*p* = 0.564), abnormal oocyte (*p* = 0.827), oocytes metaphase I and II (*p* = 0.306; *p* = 0.165), germinal vesicles (*p* = 0.076), grade I, II, and III fertilized oocytes (*p* > 0.05), and for maturation rate (*p* = 0.13). There were also no significant differences (*p* > 0.05) in embryo quality parameters with the mean number of grade I, II, and III fertilized oocytes and the fertilization rate, the number of transferred embryo (0.086). There were no significant differences between vaccinated and unvaccinated groups with respect to follicular fluid SARS‐CoV‐2‐IgG (*p* = 0.854), and pregnancy outcomes (*p* = 0.550).

**Conclusions:**

The COVID‐19 mRNA vaccine has no effect on ICSI, fertility parameters, and pregnancy outcome.

## 1 INTRODUCTION

COVID‐19 started in Wuhan, the Hubei province of China (D99‐1), in December 2019 and rapidly spread worldwide.^1^ Millions of people were affected with COVID‐19‐related illness, with mortality rates exceeding severe acute respiratory syndrome and Middle East respiratory syndrome combined.^2^ By 2021, vaccine drives against COVID‐19 were underway worldwide.^3^ During November 2021, the World Health Organization (WHO), in partnership with the Iraqi Ministry of Health, launched a national COVID‐19 mass vaccination campaign covering all of Iraq, including the Kurdistan region.^4^ There were three vaccines approved for use in Iraq by 2022: the mRNA vaccine (Pfizer BioNTech); ChAdOx1 nCoV‐19 (AstraZeneca/Oxford), and the inactivated SARSCoV‐2 vaccine, BBIBP‐CorV (Sinopharm).^5^ However, most vaccinated Iraqis have received the freely available Pfizer‐BioNTech mRNA COVID‐19 via government funding agreements. The Pfizer vaccine produces high SARS‐CoV‐2 neutralizing antibody titers together with antigen‐specific CD8+ and Th1type CD4+ cells for providing immunity.^6^ Two doses of the Pfizer BioNTech confer 95% protection against COVID‐19 infection with a favorable safety profile within 2 months of median follow up.^7^

There is significant COVID‐19 vaccine hesitancy among the Iraqi population.^8^, ^9^ Studies show that anxiety about adverse events and vaccine efficacy, and misinformation about the COVID‐19 vaccines via social media, are causing vaccine hesitation.^5^, ^10^ On news outlets and social media platforms in Iraq, many claims have been raised regarding the deleterious effects of COVID‐19 vaccines on sperm quality without scientific evidence.^11^ However, a recent prospective observational study in Iraq demonstrated that the Pfizer‐BioNTech mRNA COVID‐19 vaccine has no deleterious effects on semen parameters.^11^ There is still a paucity of information on the impact of COVID‐19 vaccines on other fertility outcomes, including assisted reproductive techniques of vitro fertilization (IVF) or intracytoplasmic sperm injection (ICSI) treatment. One study in a female Israeli population, showed the Pfizer BioNTech (BNT162b2) vaccine does not compromise IVF performance and outcomes from the early stage of oocyte development through to the early beginning of pregnancy.^12^ Similar findings were reported in China.^13^ Another retrospective study found that different types of COVID‐19 vaccines had no effect on IVF outcomes in Jordanian women.^14^ An international meta‐analysis also found no scientific proof of any association between COVID‐19 vaccines and fertility impairment in men or women.^15^

The updated ASRM guidelines also consider it unlikely that gametes or embryos would be affected by SARS‐CoV‐2 infection.^16^ A recent study confirmed that COVID‐19 infection did not affect patients' performance or ovarian reserve in the immediate subsequent IVF cycle, except for a reduced proportion of top‐quality embryos^6^ In agreement with the ASRM, previously we also determined that infection levels of SARS‐CoV‐2 IgG in follicular fluid, had no effect on embryo and oocyte quality for ICSI.^17^ This current study aimed to follow up the same cohort to investigate fertility parameters and pregnancy outcome from ICSI in relation to the Pfizer‐BioNTech mRNA COVID‐19 vaccine. We investigated ovarian stimulation characteristics, together with oocytes and embryo quality, in patients undergoing the ICSI procedure at a fertility clinic. The findings of this study will be used to help fertility specialists counsel patients regarding the vaccination decision process as well as ICSI after vaccination and will have wider benefits for public health programs initiated in Iraq.

## 2 METHODS

### 2.1 Study population

**Figure 1:**
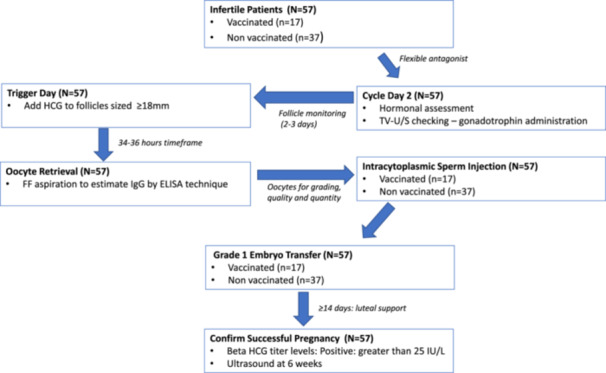
Study flow chart.

The prospective cohort study was performed over a 1 year period between January 2021 and December 2022. The study population included all couples undergoing ICSI infertility treatment. This study enrolled 54 patients who had reached the stage of ovum pick‐up, including 17 vaccinated and 37 non‐vaccinated, as shown in Figure 1. Vaccinated patients received two doses of the Pfizer vaccine and reached the stage of ovum pick‐up. All patients received full doses over varying periods before ova pick up. The vaccination interval before ova pick‐up was classified into: “<90 days, 90–180 days and >180 days.”

#### 2.1.1 Inclusion criteria

1. Patients aged 18–40 years.
2. Patients recovered from COVID‐19 infections with no acute infection, as documented by negative PCR tests.
3. Patients vaccinated by Pfizer‐BioNTech COVID‐19 vaccine as a full dose vaccine.
4. Day 3 grade I embryos.

#### 2.1.2 Exclusion criteria

1. Old women (aged > 40).
2. Young women (aged < 18).
3. COVID‐19 IgM+ cases (acute infection).
4. Poor ovarian responder (<3 follicles).
5. Endometriosis (mild, moderate, and severe).
6. Overt medical disease, including thyroid dysfunction, diabetes mellitus, and hyperprolactinemia.
7. Severe oligospermia, asthenozoospermia, and teratoazoospermia (OAT).

### 2.2 Infertility protocol

#### 2.2.1 Preparation for controlled ovarian stimulation (COS) in patients

To prepare for COS, baseline levels of luteinizing hormone (LH), follicle‐stimulating hormone (FSH), estradiol, and progesterone were assessed in each patient, and transvaginal ultrasonography was performed before gonadotropin hormone administration. The starting dose of gonadotropin hormone was chosen depending on the patient's age, body mass index (BMI), baseline FSH concentration, follicle count, and anti‐Müllerian hormone (AMH) concentration.

The flexible antagonist protocol for IVF/ICSI cycle stimulated the ovaries of each patient. Transvaginal ultrasound was performed to exclude the presence of ovarian cysts and to measure endometrial thickness. The protocol started on Day 2 provided that estadiol‐2 levels were <50 pg/mL and endometrial thickness was <5 mm. Recombinant FSH was injected subcutaneously daily at 150–300 international units (IU). The first ultrasound scan was undertaken on Day 5, and thereafter 2–3 days apart. Serum estradiol‐2 was assessed on Days 6–8 during Gonal‐F injection, a medicine that contains active follitropin alfa, and until the day of triggering. The gonadotropin‐releasing hormone (GnRH) antagonist used was daily injectable Cetrorelix (0.25 mg) provided intramuscularly when the dominant follicles reached a size of 12–14 mm. The GnRH antagonist and Gonal‐F regime continued concomitantly until the development of a dominant follicle the size of 17–18 mm. Triggering of ovulation used recombinant human chorionic gonadotropin (rhCG) (1000–5000 IU) or ovidriel (250 μg) subcutaneously, as reported by Hou et al.^18^

#### 2.2.2 Oocyte retrieval

Each anesthetized patient had their oocytes aspirated 34–36 h following oocytes triggering, under transvaginal ultrasound, in the operating theatre (Appendix S1).

Follicular fluid was aspirated from follicles of each patient on the day of ovum pick‐up. Only macroscopically clear fluid was utilized to guarantee a lack of contamination or blood. Immediately following oocyte retrieval, follicular fluid was subject to centrifugation at 3000 rpm for 20 min to remove debris. The fluid was transferred to a sterile polypropylene tube, are the supernatant picked up and stored at −20°C until assayed.

#### 2.2.3 Oocyte assessment

The following parameters were used for assessing oocytes: germinal vesicle stage (GV), metaphase I (MI) oocytes, abnormal oocytes,^19^ and the maturation rate (estimated by dividing number of oocytes on total oocytes). A low maturation rate was considered at <75%, and normal maturation at ≥75%.^20^

#### 2.2.4 Embryo quality assessment

Embryo quality following the ICSI procedure was assessed as follows: grade 1 embryo (good), grade 2: (fair), grade 3 (poor), and fertilization rate as calculated ([number of two pronuclei (2PN)/total number of M II retrieved oocytes] * 100). Fertilization is a strong, independent predictor of implantation rate and may be useful in modeling to guide decision‐making for the number of embryos to transfer.^21^ Both high fertilization (>50%) and low fertilization rates (≤50%) were considered as reported by Rosen et al.^21^ (refer to Appendix S1 for detailed protocol). The embryos were graded with respect to morphological criteria. Good Day 3 embryos had at least 6–10 cells and less than 20% fragmentation. The best embryo was transferred (grade 1). Patients were provided with progesterone supplements (60 mg, once daily, intramuscularly) to support the luteal phase. Pregnancy was documented by positive serum beta hCG at a level of 6.5 IG/mL 2 weeks after embryo transfer. Clinical pregnancy was confirmed in the 6th week using ultrasound detection.

#### 2.2.5 ELISA detection of SARS‐CoV‐2 IgG

Follicular fluid SARS‐CoV‐2 IgG was measured using the enzyme‐linked immunosorbent assay (ELISA) Elabscience kit according to the manufacturer's instruction for SARS‐CoV‐2.^22^ Patients with follicular fluid SARS‐COV‐2 IgG were categorized into three groups: low (SARS‐CoV‐2 IgG < 0.6 IG/mL), medium (SARS‐CoV‐2 IgG 0.6–1 IG/mL) and high (follicular fluid SARS‐CoV‐2 IgG > 1 IG/mL) (Appendix S2).

### 2.3 Statistical analysis

The “guidelines for reporting of statistics for clinical research in urology” were used to inform the analysis, reporting, and interpretation of the research study, as reported by Assel et al.^23^ Data were described in the 54 patients entering the ICSI Protocol using frequency and percentage for qualitative variables while the mean and standard deviation adopted for quantitative variables. The normality of the data was estimated using Kolmogorov and Smirnov tests and skewness and elongation indices. Two‐sided independent Student *t*‐tests and analysis of variance compared means in numerical variables. The Pearson's chi‐squared and Fisher's exact test analyzed categorical variables. The Fisher's exact test described any frequency in the contingency table of less than 5, while Pearson's chi‐squared was used as the default test for categorical variables analyses. Non‐parametric testing used the Mann–Whitney *U* Test. Data analysis was performed using IBM SPSS Statistics (Version 26)^24^ at a significance level α = 0.05.

## 3 RESULTS

### 3.1 Patient characteristics

Table 1 reports the patient characteristics, that comprised fewer vaccinated (*n* = 17) compared to non‐vaccinated (*n* = 37) cases. Across both study groups, most patients were under 35 years old, with a high BMI (>30) noted in a proportion of the unvaccinated groups (40.5%). There were no significant differences regarding the age and BMI ranges (*p* = 0.965, *p* = 0.454) between patient groups. A high cause of infertility related to male factors (*n* = 16, 43.2%) and polycystic ovary syndrome (n = 13, 35.1%) in the unvaccinated group and unvaccinated patients (*n* = 4, 23.5%; *n* = 5, 29.4%). The collective causes of fertility neared a significant difference between the vaccinated and unvaccinated groups (*p* = 0.09). However, there were no significant differences between groups with respect to infertility type (primary or secondary) and infertility duration (*p* > 0.05). Both groups were well matched for hormone levels of estradiol at the day of trigger (1085.82 ± 975.25, 923.42 ± 595.61; *p* = 0.50) or the second cycle day (31.79 ± 10.756, 41.78 ± 26.90; *p* = 0.16). Levels were also well matched among study groups for AMH (1.46 ± 1.15, 1.40 ± 1.05; *p* = 0.75), FSH (5.96 ± 3.77, 6.44 ± 3.16; *p* = 0.65), LH (4.62 ± 2.75, 4.20 ± 1.59; *p* = 0.60), and prolactin (18.65 ± 7.358, 24.16 ± 16.82; *p* = 0.21).

**Table 1:** Patient demographics, fertility parameters, and hormone levels.

Parameters	Non‐vaccinated (*n* = 37)	Vaccinated (*n* = 17)	*p*‐Value
Age range (years)^b^ *n* (%)			
<35	22 (59.4%)	10 (58.8%)	0.965
35–40	15 (40.5%)	7 (41.1%)	
BMI^b^ *n* (%)			
<25	9 (24.3%)	6 (35.3%)	0.454
25–30	13 (35.1%)	7 (41.1%)	
>30	15 (40.5%)	4 (23.5%)	
Occupation^b^ *n* (%)			
Housewife	31 (83.8%)	15 (88.2%)	0.509
Employed	6 (16.2%)	2 (11.8%)	
Type of infertility^b^ *n* (%)			
Primary	28 (75.7%)	13 (76.5%)	0.617
Secondary	9 (24.3%)	4 (23.5%)	
Infertility cause^b^ *n* (%)			
Male factor	16 (43.2%)	4 (23.5%)	0.089
PCOS	13 (35.1%)	5 (29.4%)	
Tubal	0 (0.0%)	2 (11.8%)	
Unexplained	8 (21.6%)	6 (35.3%)	
Mean (± standard deviation) patient reporting			
Duration of infertility^b^	8.14 ± 5.396	7 ± 4.486	0.454
Estadiol‐2: trigger day^a^	1085.820 ± 975.248	923.419 ± 595.607	0.530
AMH^a^	1.485 ± 1.150	1.379 ± 1.050	0.747
FSH^a^	5.962 ± 3.771	6.444 ± 3.158	0.649
LH^a^	4.629 ± 2.749	4.207 ± 1.590	0.593
PRL^a^	18.647 ± 7.358	24.161 ± 16.828	0.211
Estradiol: cycle day 2^a^	31.798 ± 10.756	41.7818 ± 26.90211	0.157

Abbreviations: AMH, anti‐Müllerian hormone; FSH, follicle stimulating hormone; LH, luteinizing hormone, PCOS: polycystic ovary syndrome; PRL, prolactin.

^a^ Independent samples Student's *t*‐test.

^b^ Chi‐squared test.

### 3.2 Oocyte and embryo quality, fertilization, and pregnancy rates

As shown in Table 2 (first row), there was a similar number of picked‐up oocytes (per patient) in each study group (*p* = 0.56), ranging from 11.08 ± 5.87 to 10.18 ± 3.78. The number of mature oocytes (metaphase II stage) suitable for the ICSI procedure was high in both patient groups (7.222 ± 4.47, 5.53 ± 3.06) when compared to immature oocyte numbers (1.83 ± 1.20, 2.27 ± 0.91). There were no significant differences in metaphase II mature oocytes (7.22 ± 4.47, 5.53 ± 3.06; *p* = 0.170) between the vaccinated and unvaccinated groups. However, the mean number of grade I embryo transfers was almost significantly greater in the non‐vaccinated patient (2.14 ± 0.915 vs 1.64 ± 0.50) group (*p* = 0.066), as with the number of transferred embryos. Although a high fertilization rate (>50%) was also observed in non‐vaccinated patients (*n* = 30 out of 37 patients), there were no significant differences in pregnancy outcome among the study groups (*p* = 0.550).

**Table 2:** Fertility parameters in patient groups (including oocyte and embryo quality), key indicators of pregnancy, and SARS‐Cov‐2 IgG levels.

Fertility parameters		Non‐vaccinated (*n* = 37)	Vaccinated (*n* = 17)	*p*‐Value
Mean number (+/−SD) per patient				
Picked up oocytes		11.081 ± 5.866	10.176 ± 3.779	0.564
Abnormal oocytes		2.750 ± 4.669	2.429 ± 2.299	0.827
Immature oocytes metaphase I		1.833 ± 1.200	2.273 ± 0.905	0.306
Mature oocytes metaphase II		7.222 ± 4.473	5.529 ± 3.064	0.165
Germinal vesicles		1.944 ± 0.639	3.111 ± 1.691	0.076
Grade I fertilized oocyte		2.553 ± 1.644	2.352 ± 1.156	0.652
Grade II fertilized oocyte		1.876 ± 1.032	2.143 ± 0.378	0.512
Grade III fertilized oocyte		2.300 ± 1.889	2.000	0.883
Grade I embryo transfer		2.137 ± 0.915	1.642 ± 0.497	0.066
Transferred embryos		2.179 ± 0.905	1.643 ± 0.497	0.086
Number of patients (%)				
Fertilization rate ‐ low (≤50%)		7 (18.9%)	6 (35.3%)	0.303
Fertilization rate ‐ high (>50%)		30 (81%)	11 (64.7%)	
Nonpregnant outcome		27 (72.9%)	12 (70.6%)	0.550
Pregnant outcome		10 (27.0%)	5 (29.4%)	
Follicular fluid SARS‐Cov‐2 IgG/mL	Low (<0.6)	3 (8.10%)	2 (11.8%)	0.854
	Medium (0.6–1)	3 (8.10%)	1 (5.88%)	
	High (>1)	31 (83.8%)	14 (82.4%)	

Abbreviations: IgG, immunoglobulin G; SARS‐CoV‐2, severe acute respiratory syndrome coronavirus 2.

**Figure 2:**
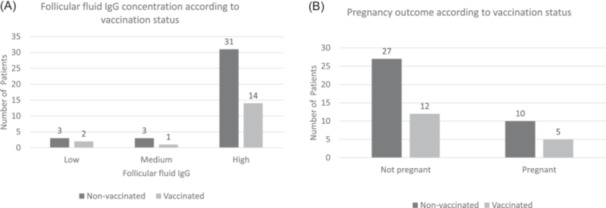
Clustered bar chart showing: (A) SARS‐CoV‐2 follicular fluid IgG in the studied groups. (B) Pregnancy outcomes in the studies groups. IgG, immunoglobulin G; SARS‐CoV‐2, severe acute respiratory syndrome coronavirus 2.

Furthermore, there are no significant differences between non‐vaccinated and vaccinated groups for embryo quality parameters: number of transferred embryos, fertilization rate, mean grade I fertilized oocyte, mean grade II fertilized oocyte, and mean grade III fertilized oocyte (Table 2). The level of follicular fluid IgG was higher in the non‐vaccinated patients compared to vaccinated, most notably in the high category (Figure 2A). Across the follicular fluid SARS‐Cov‐2 IgG groups, there were no significant differences (*p* = 0.854). A similar proportion of non‐vaccinated (72.9%) and vaccinated women (70.6%) did not obtain a positive pregnancy outcome and 5 (Table 2, Figure 2B showing patient numbers). There was no significant difference across the pregnancy groups according to vaccination status (*p* = 0.550).

### 3.3 Effect of vaccination interval on fertility parameters

**Figure 3:**
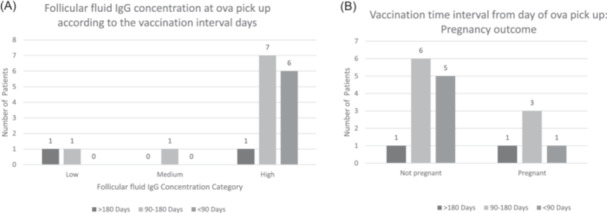
Clustered bar chart showing: (A) follicular fluid IgG in the vaccinated groups with respect to vaccination time interval. (B) Clustered bar chart showing pregnancy outcomes in the vaccinated group with respect to vaccination time interval. IgG, immunoglobulin G.

The vaccination interval (Table 3) had no effect on oocytes quality parameters: picked up oocytes (*p* = 0.732), abnormal oocytes (*p* = 0.688), germinal vesicles (*p* = 0.136), immature oocytes metaphase I (*p* = 0.672), and mature oocytes in metaphase II (*p* = 0.347). There was no significant difference in the follicular fluid IgG concentration category (low, medium, or high) measured at ova pick up according to the vaccination time interval at 180 days, 90–180 days, or <90 days (*p* = 0.510) as shown in Table 3, and Figure 3A. To note the high levels of SARS‐Cov‐2 IgG in non‐vaccinated patients can be caused by prior asymptomatic or subclinical infection.

**Table 3:** Vaccination interval from day of ova pick up in the studied groups.

Parameters		Non‐vaccinated	Vaccination interval from day of ova pick up				*p*‐Value
			<90 days (short)	90–180 days (intermediate)	>180 days (long)	Total % across groups	
Maturation index	Poor (<75%)	23 (62.2%)	4 (10.8%)	8 (21.6%)	2 (5.4%)	100	0.415^a^
	Normal (≥75%)	14 (82.4%)	2 (11.8%)	1 (5.9%)	0 (0.0%)	100	
Fertilization index	Low (≤50%)	7 (53.8%)	3 (23.1%)	6 (23.1%)	0 (0.0%)	100	0.293^c^
	High (>50%)	30 (73.2%)	3 (7.3%)	6 (14.6%)	2 (4.9%)	100	
Follicular fluid SARS‐CoV‐2 IgG	Low (<0.6)	3 (60.0%)	0 (0.0%)	1 (20.0%)	1 (20.0%)	100	0.510
	Medium (0.6–1)	3 (75.0%)	0 (0.0%)	1 (25.0%)	0 (0.0%)	100	
	High (>1)	31 (68.9%)	6 (13.3%)	7 (15.6%)	1 (2.2%)	100	
Age group	≤35 yrs.	22 (68.8%)	5 (15.6%)	5 (15.6%)	0 (0.0%)	100	0.282^c^
	>35 yrs	15 (68.2%)	1 (4.5%)	4 (18.2%)	2 (9.1%)	100	
BMI	<25	9 (60.0%)	2 (13.3%)	4 (26.7%)	0 (0.0%)	100	0.662^c^
	25–30	13 (65.0%)	2 (10.0%)	4 (20.0%)	1 (5.0%)	100	
	>30	15 (78.9%)	2 (10.5%)	1 (5.3%)	1 (5.3%)	100	
Pregnancy outcomes	Nonpregnant	27 (69.2%)	5 (12.8%)	6 (15.4%)	1 (2.6%)	100	0.714^c^
	pregnant	10 (66.7%)	1 (6.7%)	3 (20.0%)	1 (6.7%)	100	
Number of picked up oocytes (*N* = 54)		11.08 ± 5.87	9.17 ± 3.66	11.33 ± 3.81	8.00 ± 4.24		0.732
Abnormal oocytes (*N* = 23)		2.75 ± 4.67	1.00 ± 0.00	2.00 ± 1.41	7.00 ± 0.00		0.688
Germinal vesicles (*N* = 27)		1.94 ± 0.64	2.67 ± 1.53	3.33 ± 1.86	0.00		0.136^b^
Embryo transfer (*N* = 54)		2.78 ± 1.11	1.83 ± 0.98	2.56 ± 1.01	2.00 ± 0.00		0.201
Immature oocytes metaphase I		1.83 ± 1.20	2.00 ± 1.00	2.50 ± 0.84	2.00 ± 1.41		0.672
Mature oocytes metaphase II		7.22 ± 4.47	6.50 ± 3.39	5.56 ± 2.92	2.50 ± 0.71		0.347
grade I fertilized oocyte		2.55 ± 1.64	1.91 ± 0.80	2.61 ± 1.41	2.50 ± 0.71		0.809
grade II fertilized oocyte		1.88 ± 1.03	2.50 ± 0.71	2.00 ± 0.00	0.00 ± 0.00		0.664
grade III fertilized oocyte		2.30 ± 1.89	2.00 ± 0.0	0.0 ± 0.00	0.0 ± 0.00		0.883
Endometrial thickness at the day of ova pick up		8.26 ± 1.78	8.62 ± 1.53	7.56 ± 1.21	9.20 ± 1.13		0.484

Abbreviations: IgG, immunoglobulin G; SARS‐CoV‐2, severe acute respiratory syndrome coronavirus 2.

^a^ There are significant differences between those <90 with those 90–180 and >180 groups only.

^b^ Mann–Whitney *U* test.

^c^ Fisher's exact test.

Likewise, embryo quality was unaffected by the vaccination interval by comparing the following parameters; mean number of grade I fertilized oocyte (*p* = 0.809), mean number of grade II fertilized oocyte (*p* = 0.664), mean number of grade III fertilized oocyte (*p* = 0.883), and mean number of embryo transfer (*p* = 0.201). The fertilization rate was divided into low (≤50%) and high (>50%) and was not affected by the vaccination interval (*p* = 0.293).

### 3.4 Pregnancy outcomes in correlation to vaccination interval (Table 3) and (Figure 3B)

The failure of women to get pregnant was observed across non‐vaccinated cases (*n* = 27) at a short interval (*n* = 5), intermediate interval (*n* = 6) and long interval (*n* = 1). Pregnancy was achieved in 10 non‐vaccinated women: one woman with a short vaccination interval, three women with intermediate vaccination interval, and one woman with long time interval. There were no significant differences between groups (*p* = 0.714) (Table 3 and Figure 3B).

### 3.5 Hormonal profile and endometrial thickness in correlation to vaccination interval

The mean hormonal levels of AMH, FSH, luteinizing hormone, prolactin, and estradiol were reported with respect to the vaccination interval (Table 4). There was no significant difference in hormonal levels and endometrial thickness at the day of ova pick up when correlated to the vaccination interval in the studied groups (*p* > 0.05) (refer to Tables 3 and 4).

**Table 4:** Time interval from day of vaccination to ova pick up in the vaccinated group and hormonal levels.

Mean ± standard deviation parameters	Vaccination period from day of ova pick up (*N* = 17)			*p*‐Value
	<90 days	90–180 days	>180 days	
Estradiol at ova pick up	8.617 ± 1.530	7.556 ± 1.206	9.200 ± 1.131	0.186
Estradiol	732.183 ± 379.321	1143.773 ± 664.431	505.535 ± 647.377	0.255
AMH	1.163 ± 0.310	1.707 ± 1.342	0.550 ± 0.354	0.324
FSH	6.722 ± 5.164	5.981 ± 1.576	7.695 ± 0.912	0.782
LH	3.598 ± 1.844	4.338 ± 0.887	5.640 ± 2.348	0.302
Prolactin	25.302 ± 7.695	25.687 ± 22.300	13.870 ± 4.002	0.683
Estradiol at second ovulation day	34.865 ± 14.157	49.200 ± 33.876	29.150 ± 17.183	0.497

Abbreviations: AMH, anti‐Müllerian hormone; FSH, follicle stimulating hormone; LH, luteinizing hormone.

## 4 DISCUSSION

COVID‐19 disease is an alarming global health problem that affects all aspects of life and healthcare systems.^2^ The use of assisted reproductive techniques such as ICSC to address fertility is not exempted by SARS‐CoV‐2 infection. COVID‐19 infection is associated with the release of many cytokines that elicited a sustained systemic inflammatory response,^25^ and causes a significant reduction in the proportion of top‐quality embryos from IVF cycles.^6^ During the pandemic, the European Society of Human Reproduction and Embryology issued recommendations to temporarily suspend fertility services.^26^

New COVID‐19 variants are still spreading fast among populations worldwide,^27^ and preventive measures that contain new waves within countries such as Iraq are essential.^28^ Vaccination remains an important measure to prevent serious infection, and public misinformation and doubts regarding the vaccines should be properly addressed, to guide populations into safer, informed choices and provide clinicians with evidence based, scientific information.^15^ However, the lack of scientific data regarding the health effects of mRNA vaccines on fertility and assisted reproductive techniques‐related outcomes, specifically in the Iraq population, is contributing to a lack of confidence in vaccines, and hence the need for the current investigation. There have been unfounded claims in the Iraq media blaming the SARS‐CoV‐2 vaccine as a potential cause of sterility. These unscientifically unproven reports have led to hesitancy among women to take the COVID‐19 vaccine, especially among reproductive age groups. One proposed mechanism for this belief is that there is a supposed similarity between synectin‐1 and CoV‐2 spike protein,^29^ that may induce immune cross‐reactivity that hinders the developing embryo, resulting in female sterility. However, laboratory analyses have failed to prove any evidence of cross reactivity, seropositivity to the SARS‐CoV‐2 spike protein, whether from vaccination or infection, does not prevent embryo implantation or early pregnancy development.^29^

This study aimed to clarify the health effects of the mRNA COVID‐19 vaccine on fertility parameters during and after ICSC procedures. In agreement with previous studies in other countries,^12^, ^13^, ^15^ we found there was no influence of the mRNA SARS‐CoV‐2 vaccine on patients' performance during ICSI, and no detrimental effects were observed on ovarian reserve, oocytes, and embryos quality. There were no significant differences between vaccinated and unvaccinated groups with respect to follicular fluid IgG (*p* = 0.854), and pregnancy outcomes do not differ (*p* = 0.550). The pregnancy outcomes were within the accepted range (17 out of 54 i.e., 29.4% per transfer). Similar reports are stated by others,^12^, ^13^, ^15^ who found that mRNA SARS‐CoV‐2 vaccine did not affect ovarian response as well as treatment outcome in IVF‐treated patients.^30^ Our study also noted no significant effect of vaccination on endometrial thickness (*p* = 0.484). The same result was reported by Morris (2021), who found that endometrial thickness displays no significant difference between SARS‐CoV‐2 seronegative women and seropositive ones, whether due to vaccination or infection.^29^ The lack of effect of the mRNA SARS‐CoV‐2 vaccine on ovarian stimulation characteristics, oocyte and embryo quality, and pregnancy outcome suggests that the immune response elicited to the vaccine does not compromise female fertility.

In summary, the present study concludes that:

1. mRNA SARS‐CoV‐2 vaccine has no effect on ovarian sex hormone production.
2. Ovarian reserve (as measured by AMH) shows no significant alteration in patients after mRNA SARS‐CoV‐2
3. mRNA SARS‐CoV‐2 does not affect embryo and oocytes quality in patients undergoing ICSI.
4. The SARS‐COVID‐19 vaccine does not affect pregnancy outcome in assisted reproductive techniques procedures.
5. Time interval after vaccination shows no significant impact on pregnancy outcome in ICSI.
6. Pregnancy outcome does not figure out any significant difference in relation to mRNA SARS‐CoV‐2 and anti‐SARS‐COVID‐19 IgG levels.
7. The myth of association between the SARS‐COVID‐19 vaccine and sterility is unfounded.
8. Public health doctors in Iraq can counsel women of reproductive age that seropositivity for SARS‐CoV‐2 does not associate with infertility and does not interfere with embryo development.

### 4.1 Limitations and future studies

A limitation of the current study is the sample size, although convenient, studies with larger sample size will provide more robust conclusions. However, conflicts raised by the sample size were addressed by using the appropriate statistical test. A further limitation is that long‐term health effect of the vaccine on fertility parameters has not been assessed. The strength of the current study is that it is designed to investigate the potential effect of vaccination on assisted reproductive techniques protocols to participate with other authors to build guidelines in the era of the SARS‐COVID‐19 pandemic. The current study will also follow up on the health of pregnant women to determine if there are long‐term health effects of mRNA vaccinations. Future studies will also explore the fertility effects of any new types of COVID‐19 vaccines administered in Iraq (mRNA or protein‐based vaccines).

## AUTHOR CONTRIBUTIONS

**Hawraa Sahib Al‐Haddad**: Conceptualization; formal analysis; investigation; Methodology; data curation; writing—original draft; software; project administration. **Hayder A. L. Mossa**: Project administration. **Amal Abdulwahid Muhammed**: Project administration; resources. **Alaa Salah Jumaah**: Conceptualization; investigation; writing—original draft; methodology; data curation; formal analysis; software. **Katherine Ann McAllister**: Conceptualization; writing—original draft; writing—review & editing; project administration. **Akeel Abed Yasseen**: Writing—original draft; project administration; resources; supervision; conceptualization; formal analysis.

## CONFLICTS OF INTEREST STATEMENT

All authors have completed the ICMJE uniform disclosure form. The authors declare no conflicts of interest.

## ETHICS STATEMENT

The authors are accountable for all aspects of the work in ensuring that questions related to the accuracy or integrity of any part of the work are appropriately investigated and resolved. All procedures performed in studies involving human participants were in accordance with the ethical standards of the institutional and/or national research committee(s) and with the Helsinki Declaration (as revised in 2013). This study was approved by the institution research committee at the High Institute for Infertility Diagnosis and Assisted Reproductive Technologies/Al‐Nahrain University, IRB (2/3/13008). Informed consent had been taken from all patients enrolled in the study. All the authors are responsible for any false statements or failure to follow the ethical guidelines.

## TRANSPARENCY STATEMENT

The lead author, Katherine Ann McAllister, affirms that this manuscript is an honest, accurate, and transparent account of the study being reported; that no important aspects of the study have been omitted; and that any discrepancies from the study as planned (and, if relevant, registered) have been explained.

## Supporting information

Supporting information.

Supporting information.

## Data Availability

The data that support the findings of this study are available on request from the corresponding author. The data are not publicly available due to privacy or ethical restrictions.
