# Supplementary material for: The impact of SARS‐CoV‐2 mRNA vaccine on intracytoplasmic sperm injection outcomes at a fertility center in Iraq: A prospective cohort study
Source: Health Sci Rep. 2024 May 23;7(5):e2142. doi: 10.1002/hsr2.2142 (PMC11112633; doi:10.1002/hsr2.2142)
Supplement: Supplementary file 1 — Supporting information. [file HSR2-7-e2142-s001.docx]

**Supplementary appendix 1. ICSI protocol**

***Collection of Follicular fluid:***

- One milliliter of follicular fluid is aspirated at the day of ova pick up.
- The fluid is centrifuged at 3000 RPM cycle for 20 minutes.
- The Supernatant aspirated and stored at -20 °C.
- Follicular fluid IgG are investigated using ELISA immunoassay.

**Intracytoplasmic Sperm Injection (ICSI) Program:**

***Controlled Ovarian Hyper-Stimulation (COH):***

- The flexible antagonist protocol for IVF/ICSI cycle was chosen patients enrolled in the study.
- Transvaginal ultrasound was performed for all patients in order to exclude the presence of ovarian cyst and to measure endometrial thickness.
- This protocol started at day-2 provided that E2<50 pg/ml and endometrial thickness <5mm on transvaginal ultrasound.
- A recombinant human follicle stimulating hormone (rFSH) (Gonal F, Merck ® 75 IU of FSH activity per ampoules) injected subcutaneously on daily bases, the dose is 150-225 according to previous response, antral follicular count and patients’ age.
- Dose of Gonal-F and growth of follicles are monitors by transvaginal ultrasound. First ultrasound scan taken at day 5, followed by subsequent scanning 2-3 days apart as required. Serum estradiol-2 is checked on day 6-8 of Gonal-F injection and this continue till day of triggering. GnRH antagonist (Cetrorelix) (0.25 mg daily) injected intramuscularly when dominant follicles reach a size of (12-14) mm as measured by ultrasound.
- GnRH antagonist (Cetrorelix) and Gonal-F continue concomitantly until a satisfactory response reached (i.e. dominant follicle size 17-18mm).
- Triggering of ovulation done by HCG 5000-1000 IU or ovitriel 250mg subcutaneously.

***Oocyte Retrieval***

- Oocytes were aspirated under transvaginal ultrasound under general anesthesia. This is done within 34-36 hours following oocyte triggering.
- The patient is placed in the dorsal lithotomy position. The vagina is washed with normal saline. Follicles aspirated from ovaries by ova aspirated needle (Single lumen Ovum Aspiration Needle – 17 gauge and 35 cm long Cook®, Australia). Ova picked up from both right and left ovaries in order. The follicular fluid was sent to the embryo laboratory. Follicular fluid taken after obtaining informed consent agreement from patients and store at -20 for anti-COVID-19 IgG assessment.
- Retrieved oocytes-cumulus complexes are collected and then rinsed with flushing media in order to wash residual blood from the aspirate (follicular aspirate). The oocytes were graded and transferred into drops of human tubal fluid (HTF) and stored in an incubator set at 37 °C, 5%CO2, 5% O2 and 95% humidity.

***Oocytes Preparations and Assessment***

- Cumulus oocyte cells were removed by denudation. This is done by enzymatic and mechanical methods. The enzymatic method utilized hyaluronidase.
- After denudation, oocytes having normal morphology and extrude its first polar body (metaphase II) are suitable for the process of microinjection (1) (de moura *et al*., 2017). Oocytes with the following features (parameters) are excluded from the process:” amorphic shape of oocytes, granulation in the perivitelline space, presence of vacuolization, dark colored cytoplasm, in addition to the quality of zona pellucida in respect to its color and structure (2) (Thorat *et al*., 2020) .
- Germinal Vesicle Stage (GV): Refered to oocytes in the prophase of the first meiotic division.
- A Metaphase I (M I) Oocytes: Referred to oocytes depleted from germinal vesicels or first polar body in its perivitelline space.
- Abnormal Oocytes: Referred to oocytes with any of the followings:” thickened and dark colored zona pellucida, fragmented polar body, cytoplasmic inclusions, granulated perivitellline space, large cytoplasmic vacuole and large and granulated perivitelline” (3)(Yu *et al*., 2015).
- A Metaphase II (M II) Oocytes: These are oocytes having first polar body.
- Maturation Rate: maturation rate is estimated by dividing number of oocyte on total oocytes. Low maturation rate is <75% and normal maturation is ≥75% (4) (Nora *et al*., 2020).

***Intracytoplasmic Sperm Injection (ICSI) procedure***

- This is a microscopic procedure utilizing multiple micromanipulation devises (microinjectors, micropipettes and micromanipulators). A holding pipette apply gentle suctions on a stabilized mature oocytes. Here, the position of polar body is at 12 or 6 o`clock. Thin holding low glass microinjector collect a single active sperm from the opposite side. Sperm injection into oocyte follow. Sperm injected at site of 3 or 9 o`clock in mature oocytes.
- The injected oocyte cultured in CO2 incubator at 37 °C and 5%CO2. Fertilization checked next days (5) (De Vos *et al*., 2009).

***Evaluation of Fertilization and Cleavage Rates***

- Monitoring begins 18 hours after injection. On the 1st day, inverted micromicroscope used seeking for presence of two pronuclei, and zygote containing dish incubated for 3 days (Lazzaroni-Tealdi *et al*., 2015).
- Individual embryo evaluation was performed on the 3rd day under microscope before transfer. Several parameters are used to select the best embryo as size, number and quality of blastomeres, fragmentation and multinucleation degree (2) (Thorat *et al*., 2020).
- Grade 1 Embryo: (good). Stage-specific cell size, 10%-25% fragmentation and no multiniucleation.
- Grade 2: (fair). Stage-specific cell size for majority of cells, <10% fragmentation and no multiniucleation.
- Grade 3: (poor). No stage-specific cell size, ≥25% fragmentation and clear multiniucleation.
- Fertilization Rate using the following:
- Fertilization rate= [number of two pronuclei (2PN)/ total number of M II retrieved oocytes] *100. High fertilization rate (>50%) and low fertilization rate (≤50%) (6) (Rosen *et al*., 2010).

***Embryo Transfer***

- The best quality embryo is selected to be transferred (grade 1). The selected embryo is transferred to the uterus on day 3 after injection (68 cells embryo). A flexible catheter used to deliver embryo to the uterine cavity through the vagina and under abdominal ultrasound guide.
- Luteal phase support was provided with two weeks of progesterone therapy - 400mg as vaginal suppositories (Cyclogest) twice daily and until pregnancy.

***Pregnancy Test***

- Pregnancy documented by positive serum Beta hCG titer at a level of 6.5 IG/ml two weeks after embryo transfer. Progesterone treatment during first trimester.
- Clinical pregnancy sought for at 6^th^ weeks using ultrasound.

**References**:

1. Moura BR, Gurgel MC, Machado SP, et al. Low concentration of hyaluronidase for oocyte denudation can improve fertilization rates and embryo quality. *JBRA Assist Reprod*. 2017;21(1):27-30. Published 2017 Feb 1. doi:10.5935/1518-0557.20170008
2. Thorat, R.S., More, A., Salve, M.P., & Shrivastava, D. (2020). To Study the Correlation of Embryo and Oocyte Quality with Clinical Pregnancy Rate. International journal of current research and review, 12, 150-153.
3. Yu, E. J., Ahn, H., Lee, J. M., Jee, B. C., & Kim, S. H. (2015). Fertilization and embryo quality of mature oocytes with specific morphological abnormalities. Clinical and experimental reproductive medicine, 42(4), 156–162. <https://doi.org/10.5653/cerm.2015.42.4.156>.
4. Nora, H., Wiweko, B., Muharam, R., Rajuddin, Wangge, G., Hestiantoro, A., Pratama, G., Harzif, A. K., & Zakirah, S. C. (2020). Impact of Serum Human Chorionic Gonadotropin and Luteinizing Hormone Receptor Expression to Oocyte Maturation Rate: A Study of Controlled Ovarian Stimulation. Journal of human reproductive sciences, 13(1), 46–50. <https://doi.org/10.4103/jhrs.JHRS_131_19>.
5. De Vos A, Staessen C, De Rycke M, et al. Impact of cleavage-stage embryo biopsy in view of PGD on human blastocyst implantation: a prospective cohort of single embryo transfers. *Hum Reprod*. 2009;24(12):2988-2996. doi:10.1093/humrep/dep251
6. Rosen, M. P., Shen, S., Rinaudo, P. F., Huddleston, H. G., McCulloch, C. E., & Cedars, M. I. (2010). Fertilization rate is an independent predictor of implantation rate. Fertility and sterility, 94(4), 1328–1333
