# Supplementary material for: The impact of SARS‐CoV‐2 mRNA vaccine on intracytoplasmic sperm injection outcomes at a fertility center in Iraq: A prospective cohort study
Source: Health Sci Rep. 2024 May 23;7(5):e2142. doi: 10.1002/hsr2.2142 (PMC11112633; doi:10.1002/hsr2.2142)
Supplement: Supplementary file 2 — Supporting information. [file HSR2-7-e2142-s002.docx]

**Supplementary appendix 2.**

**Procedure for** **Follicular Fluid SARS-CoV-2 IgG Level Assay: Follicular Fluid CoVID-19 IgG Level Assay:**

- In this study, a total of 54 infertile women (18-40 years old) undergoing ICSI techniques are enrolled.
- The antagonist protocol was used for ovarian stimulation, consisting of gonadotropin (recombinant FSH, highly purified human menopausal gonadotropin, or a combination of both) for average of 10 days (9-12 days as a range).
- Induction of ovulation is through subcutaneous administration of GnRH agonist or hCG. Oocyte retrieval is performed under vaginal ultrasound guide 34-36 hours following induction of ovulation.
- Follicular fluid aspirated from follicles with size of 16-20 mm from each patient. A macroscopically clear fluid is exclusively chosen to avoid contamination or presence of blood. Following oocytes retrieval, follicular fluid centrifuged at 3000 RPM for 20 minutes to get rid of any debris.
- Then, fluid transferred to a sterile polypropylene tubes and the supernatant picked up, stored at - 20 ◦C till it assayed.
- Patients with follicular fluid SARS-CoV-2 IgG are divided into 3 groups as follow: low follicular fluid follicular SARS-CoV-2 IgG (follicular fluid SARS-COV-2 IgG<0.6), medium groups (follicular fluid SARS-CoV-2 IgG 0.6-1) and high group (follicular fluid SARS-CoV-2 IgG >1) (1) (Herrero *et al*.,2022)
- The level of follicular fluid SARS-CoV-2 IgG is estimated using enzyme linked immunosorbent assay (ELISA) designed for specific measurement of SARS-CoV-2 spike protein IgG.

***ELISA procedure***

- The ELISA kit used indirect ELISA as a mean for qualitative measurement to detect SARS-CoV-2 spike protein IgG in the samples. In this kit, there is micro ELISA plate which is pre-coated with purified SARS-CoV-2 Spike protein antigen (2) (Elabscience Kit for SARS-CoV-2). Following addition of samples to the well, samples SARS-CoV-2 Spike protein IgG will combine with the pre-coated SARS-CoV-2 Spike protein antigen. Following complete wash, addition of Horseradish Peroxidase (HRP) conjugated mouse anti human IgG cause development of antigen-antibody-HRP conjugated secondary antibody complex. The component which is free, washed away, and then substrate solution are added for each well. Wells that contain SARS-CoV-2 Spike protein IgG and HRP conjugated anti-human IgG will display the blue color. Stop solution are added to terminate the enzyme-substrate reaction and the color become yellow.
- The optical density (OD) is measured by spectrophotometric method at a wavelength of 450 ± 2 nm.
- Judgment for the existence of SARS-CoV-2 Spike protein IgG is by comparison with cut off value that estimated according to manufacturer guidance.
- Sample dilution: the tested fluid are diluted at 10-100 fold using control and sample diluent and mixed thoroughly.
- **Positive Control and Negative Control Working Solution**: both positive and negative control are centrifuged at 10,000×g for 1 min. we add 0.5 ml of sample and control diluent, then let it stand for a period of 10 minutes, then we invert it gently several times. Following full dissolvent, we mix it thoroughly with a pipette.
- **Concentrated HRP Conjugated Mouse Anti-human IgG Working Solution**: Calculate the required amount prior to the experiment (100μL/well). Dilute the 100×Concentrated HRP Conjugated Mouse anti-human IgG to 1× working solution with HRP Conjugate Diluent.
- **Wash Buffer:** 750mL of Wash Buffer was prepared by diluting 30mL of concentrated wash buffer with 720 mL of distilled or deionized water.
- **Assay procedures:** Determine wells for blank, positive and negative controls and for samples. Add 100μL of both samples and controls to the appropriate corresponding wells. The plates were covered by sealer provided by the Kit. Then incubate for 45 min at 37℃.
- From each well, decant the solution and add 350μL of wash buffer to each. Soak for 1-2 minutes and then we aspirate the solution from each well and pat it dry against a clean absorbent paper. The wash was repeated three times. Add 100μL of HRP Conjugated Mouse anti-human IgG working solution to each well (except the blank well). The wells covered with a plate sealer to stop evaporation and incubated at 37°C for 30 minutes.
- The solution from each well were decant and the wash was repeated for 5 times. For each well (blank well as well), we add 90μL of Substrate Reagent. Cover with a new plate sealer. Incubate for 15 minutes at 37°C. The plate should be protected from light.
- Add 50μL of Stop Solution to each well (including blank well).
- The Optical density (OD) of each well were determined at once with micro-plate reader set as 450 nm. Cut off was determined as 0.13 + negative control according to manufacturer instructions. Cut off was 0.6. The level of immunoreactivity in the patients follicular fluid was classified as low (OD<0.6), medium (OD 0.6-1) and high (OD >1).

**References:**

1. Herrero Y, Pascuali N, Velázquez C, et al. SARS-CoV-2 infection negatively affects ovarian function in ART patients. *Biochim Biophys Acta Mol Basis Dis*. 2022;1868(1):166295. doi:10.1016/j.bbadis.2021.166295
2. Elabscience Kit for SARS-CoV-2. Available at https://www.elabscience.com/p-sars_cov_2_spike_protein_igg_elisa_kit-376684.html. Last accessed on January 10, 2023.
